# An emergent infectious disease: *Clostridioides difficile* infection hospitalizations, 10-year trend in Sicily

**DOI:** 10.1007/s15010-021-01683-w

**Published:** 2021-09-08

**Authors:** Alice Annalisa Medaglia, Sergio Buffa, Claudia Gioè, Silvia Bonura, Raffaella Rubino, Chiara Iaria, Claudia Colomba, Antonio Cascio

**Affiliations:** 1 Infectious and Tropical Disease Unit, AOU Policlinico “P. Giaccone”, Palermo, Italy; 2 Dipartimento per le Attività Sanitarie e Osservatorio Epidemiologico (DASOE), Palermo, Italy; 3 Infectious Diseases Unit, ARNAS Civico, Palermo, Italy; 4 Department of Health Promotion, Mother and Child Care, Internal Medicine and Medical Specialties, Infectious Diseases Unit, University of Palermo, Palermo, Italy

**Keywords:** *Clostridioides difficile* infection, Health care-associated diarrhoea, Surveillance of *C. difficile*

## Abstract

**Background:**

*Clostridioides difficile* is the most common cause of healthcare-associated diarrhoea worldwide and *C. difficile* infection is an emerging infectious disease. In the US, its rates are monitored trough an active surveillance system, but many European Union member states still lack this, and in Italy no epidemiological data on *C. difficile* infection are available except for a few single-centre data.

**Aim:**

To provide data on the *C. difficile* infection incidence in Sicily (the biggest and 5th most populous region of Italy) during a 10-year period.

**Methods:**

We revised all the regional standardized discharge forms between 2009 and June 2019 using the code ICD-9 00845 of the International Classification of Diseases, Ninth Revision Clinical Modification, which refers to *C. difficile* infection with or without complications.

**Results:**

1139 cases of CDI were identified. 97% were adults with a median age of 73.2 years and a male-to-female ratio of 1:1.4. Female patients were older than males and patients who died were older than patients who did not. The main comorbidities were renal disease, diabetes, pneumonia and hypertension. There were 65 reporting hospitals and 86% of cases were provided by level III and II hospitals. Between 2009 and 2019, the incidence increased 40-fold. 81.5% of cases were reported in Medicine Units, Infectious Diseases Units and long-term care facilities. The mean length of stay was 20 days. Mean case fatality rate was 8.3% over the 10-year period.

**Conclusion:**

*Clostridioides difficile* infection is a dramatically increasing condition in Sicily. A high-quality surveillance system and shared diagnostic protocols are needed.

**Supplementary Information:**

The online version contains supplementary material available at 10.1007/s15010-021-01683-w.

## Introduction

*Clostridioides difficile* is the leading cause of healthcare-associated diarrhoea, often, but not always, in association with previous antibiotic use and it represents an emerging infectious disease worldwide, as its rates have dramatically increased in the last 10 years all over the world [2].

It may cause a large spectrum of disease ranging from uncomplicated diarrhoea to pseudomembranous colitis, toxic megacolon, colon perforation and multiorgan failure, resulting in significant morbidity, mortality, prolonged hospital stay and hospitalization costs [3, 4]. Main risk factors for developing *C. difficile infection* (CDI) are older age, comorbidities, long hospitalization, chronic conditions such as liver and kidney disease, inflammatory bowel disease (IBD), cancer and cancer chemotherapy, proton pump inhibitors, solid organ transplant (SOT) or haematological stem cell transplantation (HSCT) recipients, recent gastrointestinal surgery, and previous exposure to antibiotic [5].

### CDI in Northern America

In US and Northern America, CDI rates have been monitored through an active surveillance system since 2000: tracked CDI trends in these last 2 decades showed a 3.5-fold increase between 2000 and 2008 [6–9], a stable prevalence between 2011 and 2015 and an 8–12% statistically significant decrease in hospital onset CDI between 2017 and 2018 thanks to a strong surveillance system and implementation of infection control measures.

### CDI in Europe

Europe lacks an active and coordinated CDI surveillance system, but on the basis of alarming CDC data, since 2006 the European Centre for Disease Prevention and Control (ECDC) has been assessing CDI in Europe: in 2006, a CDI study involving some EU Member States was initiated to inform the scientific community and standardize diagnostic protocols [10]. In 2011, a survey of existing CDI surveillance systems was launched as part of the ECDIS-Net project (ECDC-funded “Supporting capacity building for surveillance of *C. difficile* infections) and in 2016 the ECDC coordinated data collection began [11]: 556 hospitals from 20 European countries participated and reported 7711 CDI cases in 2016, 74.6% of which were health-care related. All-cause and CDI-attributable mortality were 20.7 and 3.9%, respectively. Italy participated with just two hospitals [4]. Moreover the “European multi-centre prospective biannual point prevalence study of CDI in hospitalized patients with diarrhoea (EUCLID) reported that 25% of CDI were not diagnosed because of absence of clinical suspicion” [12].

### CDI in Italy

Italy lacks a high quality and active surveillance system for CDI, so no epidemiological data are available except for a few single-centre data [13, 14] and two recent multi-centre studies on recurrent CDI [15] and CDI underdiagnosis [16]. Indeed, in Italy CDI notification is not mandatory and infective diarrhoea by any cause (apart from Salmonella) generically falls into the category of “class 2” notifiable infectious diseases according to the Italian Superior Health Institute (ISS) notification system [17].

In 2012–2013, an Italian Centre for Diseases Control and Prevention (CCM)-financed survey, “Surveillance of Clostridium difficile infection” [18], involving 278 hospitals from 14 regions was launched but no data were disclosed or published. Likely, the CDI trend in Italy is increasing as the spread of multidrug-resistant microorganisms (MDRO), in particular of carbapenem-resistant *K. pneumoniae* (CRKP), is very concerning in this country, and antimicrobial consumption in humans is among the highest of all EU/EEA Member States, according to European Surveillance of Antimicrobial consumption network (ESAC-Net) 2015 [19]. Indeed the use of quinolones and carbapenems represents major risk factors for developing CDI [5, 20–22].

### Objectives

To provide data on *C. difficile* infection incidence in Sicily (the biggest and 5th most populous region of Italy) during a 10-year period; to provide epidemiological data on the infected population, data on distribution of cases per year, per ward, per district, on length of stay and on mortality.

## Materials and methods

We analysed a dataset provided by Regional Healthcare Department (DASOE-Dipartimento per le Attività Sanitarie e Osservatorio Epidemiologico) containing data from all the regional standardized discharged forms (SDO) from all Sicilian hospitals between February 2009 and June 2019. The SDO was established by the Italian Ministry of health in 1991 [23]: clinical and organizational information related to the hospitalization is coded using the International Classification of Diseases, 9th revision, Clinical Modification (ICD-9-CM) system [24].

To select CDI cases, the code ICD-9 00845, which refers to CDI with or without complications, was used.

We described the total number of CDI cases, mean age of total population, male to female ratio, mean age per sex, mean age in patients who died, patients’ comorbidities, and type of hospitals reporting CDI. Also, we reported the number of cases per year, per 100,000 inhabitants per year, per district per year, and per hospital, as well as the distribution of cases by ward. Furthermore, we reported the mean length of stay for all wards, for male and female patients and for fatal and non-fatal cases. Finally, we reported in-hospital all-cause mortality.

Because the data used in our analysis were completely anonymized, this study did not require approval by the institutional review board.

To calculate incidence, the number of Sicilian inhabitants surveyed through the census of 31st December 2019 was used as a denominator.

Data were analyzed using StatSoft software (Statistica™). Contingency data were analyzed by 2-tailed × 2 test or Fisher’s exact test, and continuous data by use of Student’s *t* test. The Pearson correlation coefficient was computed to verify the existence of correlations between variables. A two-sided *p* value < 0.05 was considered significant for all analyses.

Age and length of stay values were plotted by the whiskers method in which squares represent the mean values; rectangles represent mean values ± 1 standard error; and lower and upper horizontal bars correspond to mean values ± 1.96 standard error.

## Results

**Fig. 1:**
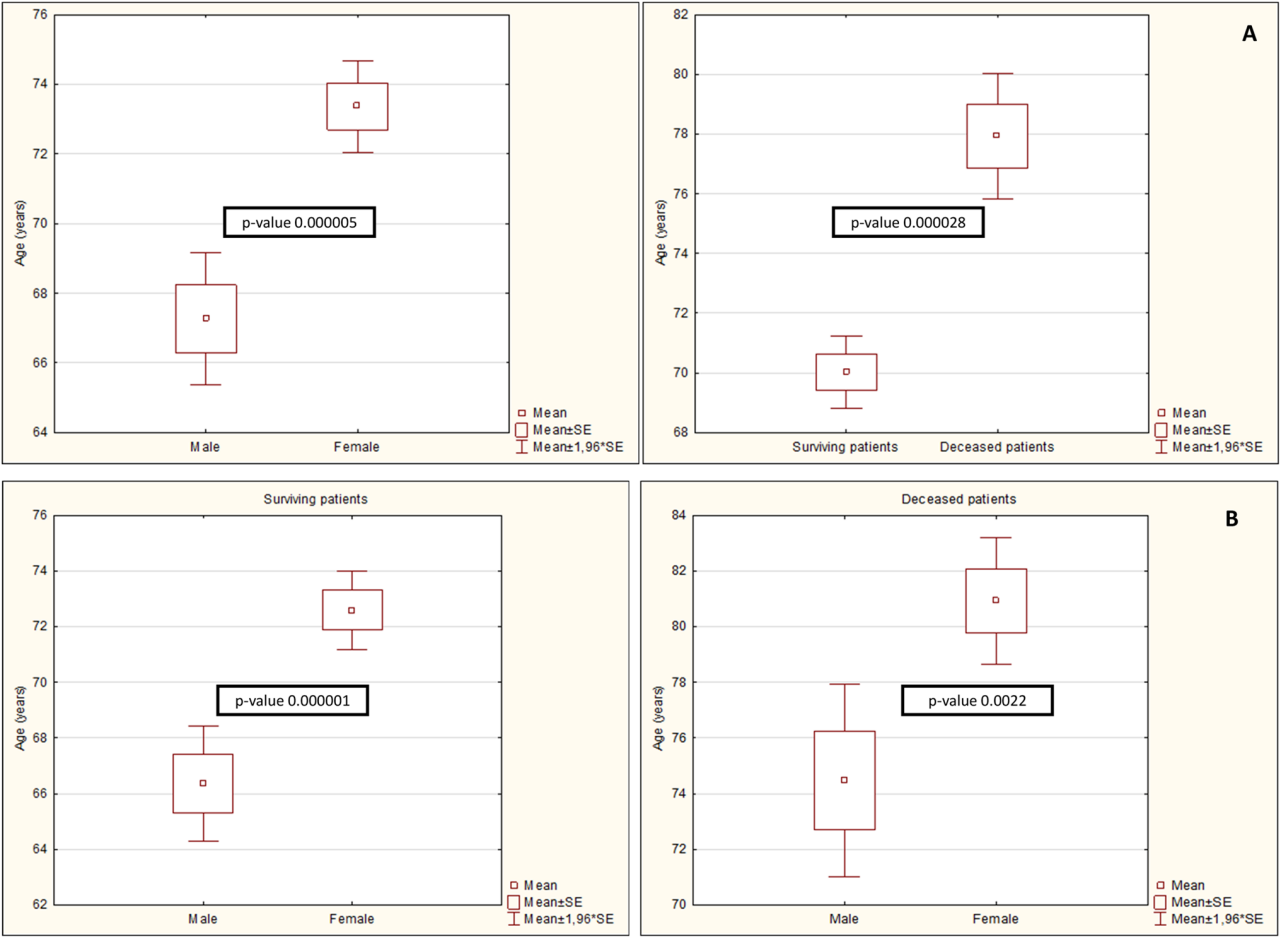
**A** On the left, difference in mean age between male and female patients; on the right, difference in mean age between patients who survived and patients who died with *C. difficile* infection. **B** On the left, difference in mean age between male and female patients who did not die; on the right, difference in mean age between male and female patients who died with CDI

During the study period in Sicily, 1139 cases of CDI were identified: 97% of CDI cases (1103 patients) were adult patients (≥ 16 years). Just 36 CDI cases were children (3%), 7 of whom < 12 months old and 3 ≤ 1 month old. The mean age of the adult patients was 73.2 years and the male to female ratio was 1:1.4. In particular, the mean age was 73.5 (± SD 17.5) and 68 (± SD 20.74) years for females and males, respectively (*p* value 0.000005); and was 77.9 (± SD 11.5) and 70 (± SD 19.9) years for patients who died with CDI and patients who did not, respectively (*p* value 0.000028). Regarding patients who did not die, the mean age was 66.3 (± SD 21.99) and 72.6 (± SD 17.84) years respectively for male and female patients (*p* value 0.000001). Regarding patients who died, the mean age was 74.5 (± SD 13) and 80.9 (± SD 17.84) respectively for males and females (*p* value 0.0022). Among male patients, 11% died (54/478 pts), and among female patients, 9% died (62/663 pts). No statistical difference was found between the percentages of deceased patients in the two sexes (Fig. 1).

Main comorbidities of patients with CDI detected during the entire decade were the following, in various combinations: renal disease in 192 patients (17% of cases), diabetes in 131 patients (11.5%), pneumonia in 127 patients (11%), hypertension in 119 patients (10%), sepsis in 94 patients (8.5%) (of whom 22 had candidemia, 27 g-negative bacteremia, 14 g-positive bacteremia, 31 not specified), cirrhosis or liver disease in 64 patients (5.6%), chronic obstructive pulmonary disease in 52 (4.5%), cancer or haematological malignancies (leukaemia, lymphoma, myeloma) in 47 (4%), shock of any kind in 46 (4%), inflammatory bowel disease in 34 (3%), fractures in 24 (2%), solid organ transplant in 19 (1.6%), tuberculosis in 15 (1.3%), myocardial infarction in 12 (1%), obesity in 6 (0.5%), urinary tract infection in 5 (0.4%), and HIV in 2 patients (0.1%).

CDI cases were registered in 65 hospitals: 47 of these were public hospitals, 18 were long-term care facilities (LTCFs) or private clinics. Of note, in Sicily there are a total of 70 public hospitals, 23 of them did not report CDI cases. Distribution by district of the 65 reporting hospitals is as follows: 20 in Catania, 14 in Palermo, 13 in Messina, 7 in Siracusa, 4 in Caltanissetta, 3 in Ragusa, 2 in Agrigento, 1 in Enna and 1 in Trapani.

18% (12/65) were level III hospitals (with 114–681 beds) which provided 49% of all cases (557/1139), 31% (20/65) were level II hospitals (with 38–425 beds), which provided 37% of cases (419/1139); 23% (15/65) were level I hospitals (with 16–130 beds), which provided 4% (44/1139) of cases, and 27% (18/65) were LTCFs, which provided 9% (104/1139) of cases.

**Fig. 2:**
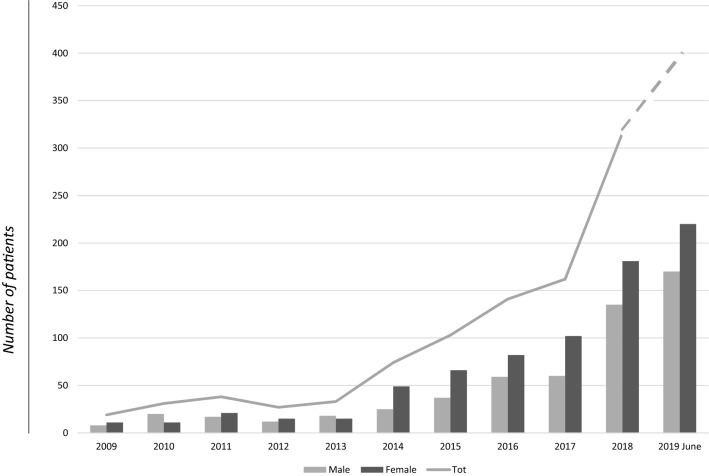
Number of reported CDI cases per year (line), and number of male vs female cases per year (columns), between February 2009 and June 2019 in Sicily. The dotted line indicates the trend for the second half of 2019

In 2009, 19 CDI cases were reported (1.7% of total cases), in 2010 31 cases (2.7% of total), in 2011 38 cases (3.3%), in 2012 27 cases (2.4%), in 2013 33 cases (2.9%), in 2014 74 cases (6.5%), in 2015 103 cases (9%), in 2016 141 cases (12.4%), in 2017 162 cases (14.2%), in 2018 316 cases (27.7%), and in 2019 195 cases (17%). See Fig. 2.

**Fig. 3:**
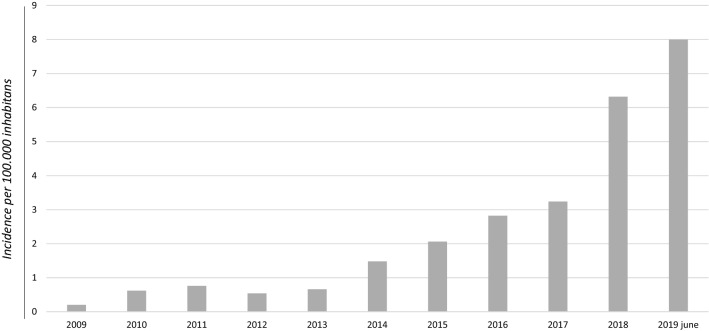
CDI incidence per 100,000 inhabitants per year between February 2009 and June 2019 in Sicily

CDI incidence per 100,000 inhabitants per year in Sicily was 0.2 in 2009, 0.6 in 2010, 0.8 in 2011, 0.5 in 2012, 0.7 in 2013, 1.5 in 2014, 2.0 in 2015, 2.8 in 2016, 3.2 in 2017, 6.3 in 2018 and 8.0 in the first half of 2019. See Fig. 3.

**Fig. 4:**
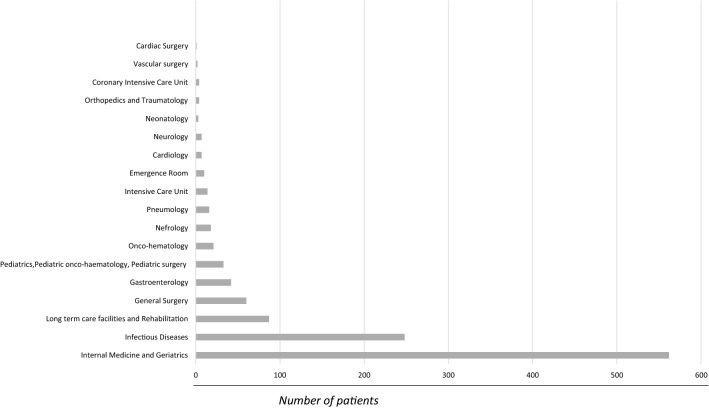
Number of CDI cases per ward in the 10-year study period in Sicily

Regarding the distribution of CDI by ward: for adult patients, 51% (562/1103) of CDI cases were reported in Internal Medicine and Geriatrics Units, 22.5% (248) in Infectious Diseases Units, 7.9% (87) in LTCFs, Rehabilitation, Neurorehabilitation and Spinal Units, 5.4% (60) in General Surgery, and 3.8% (42) in Gastroenterology. Other wards reported < 2% of total adult CDI cases. In particular, 21 cases in Hematological, Onco-hematological and Oncological Units, 18 in Nephrology, 16 in Pneumology, 14 in Intensive Care Units, 10 in Emergency Room, 7 in Cardiology, 7 in Neurology, 4 in Orthopedics, 4 in Coronary Intensive Care Unit, 2 in Vascular Surgery, and 1 in Cardiac Surgery; for pediatric patients, 33 CDI cases were reported in Pediatrics/Pediatric Onco-Haematology/Pediatric Surgery and 3 in Neonatology. See Fig. 4.

The mean length of stay, considering all the wards, was 20 days. In particular, mean length of stay was 22.9 (± SD 19.9) and 21.2 (± SD 15.7) days, respectively for male and female patients (*p* value 0.1), and was 23.7 (± SD 21.6) and 21.7 (± SD 17.06), respectively for patients who died and did not (*p* value 0.22). In both cases, a significant difference in mean length of stay between different groups was not found.

**Fig. 5:**
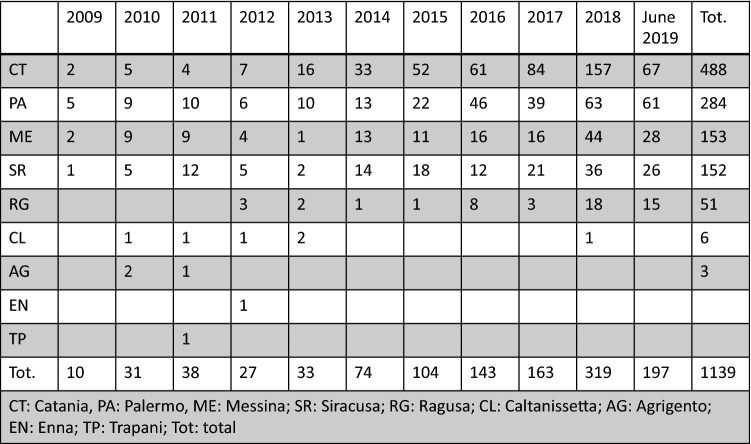
number of CDI cases per district and per year

Regarding distribution of cases in the nine Sicilian districts over the 10-year period: 43% (488) of cases were reported in Catania, 25% (284) in Palermo, 13% (153) in Messina, 13% (152) in Siracusa, 4.5% (51) in Ragusa, 0.5% (6) in Caltanissetta, 0.25% (3) in Agrigento, 0.1% (1) in Enna, and 0.1% (1) in Trapani. See Fig. 5 for details of number of cases per district per year.

Distribution of cases by hospital can be found in the supplemental material.

**Fig. 6:**
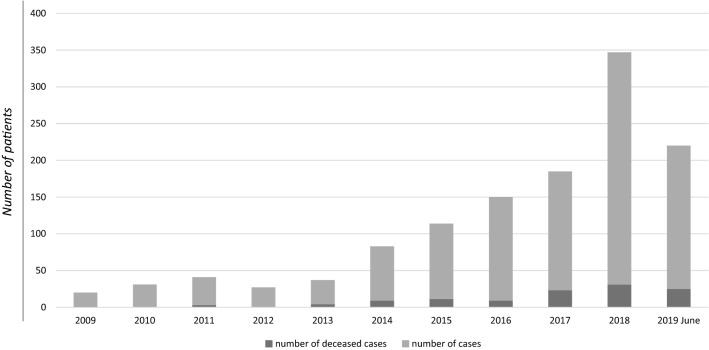
Number of total CDI cases per year and number of deceased CDI cases by any cause

Mortality in patients with CDI, considering all the wards and all the hospitals, was 5.2% in 2009, 7.9% in 2011, 12.1% in both 2013 and 2014, 10.7% in 2015, 6.4% in 2016, 14.2, 9.8 and, 12.8% respectively in 2017, 2018 and the first half of 2019. No deceased cases were registered in 2010 and 2012. See Fig. 6.

## Discussion

Retrospectively reviewing the regional hospitalization dataset referring to the period February 2009-June 2019, we found 1139 CDI cases. 97% of them were adult CDI cases. It is not specified if the cases were community acquired or healthcare associated, but all of them were reported during hospital admission.

In 2009 just 19 cases were reported vs. the 316 of 2018 and the 195 of the first half of 2019. So, the number of reported cases in 2018 was 16 times more than in 2009. Incidence per 100,000 inhabitants increased from 0.2 in 2009 to 8 in the first half of 2019, so incidence increased 40-fold. The increasing CDI trend during the decade is in accordance with worldwide data which show that CDI is an emerging infectious disease. Nevertheless, such a trend may be partially explained by increased awareness of CDI by clinicians, and thus increase in diagnosis and reporting, over the 10-year period. Regarding epidemiological characteristics of our population, the mean age, considering just adult patients ≥ 16 years old, was 73.2 and the male to female ratio was 1:1.4. This is in accordance with data provided from ECDC [11], which report a mean age of 72 years (IQR 59–80), with a slightly higher incidence among females [5].

Statistically significant differences were found in the mean age between total number of female and male patients with CDI (73.5 vs 68 years), between deceased female and male patients with CDI (80.9 vs 74.5) and between female and male patients who survived (72.6 vs 66.3). So female patients with CDI were older than males, both considering the total number of cases or just the group who died and did not. Also, patients who died were older than patients who did not die with a statistically significant difference (77.9 vs 70 years). No statistical difference was found between the percentages of dead patients between the two sexes (dead male patients/total male patients and dead female patients/total female patients).

Regarding patients’ comorbidities, which are also considered risk factors for CDI, in our population 27.1% had a chronic condition such as kidney disease, liver disease or COPD (17, 5.6, and 4.5%, respectively), 2.1% had a condition of immunosuppression (0.4% cancer or hematological malignancies, 1.6% solid organ transplant, 0.1% HIV), and 3% IBD. Even if no data are available on antibiotic use in CDI cases, 20% had a documented infection: pneumonia in 11% of cases, sepsis in 8.5%, tuberculosis in 1.3%, and urinary tract infection in 0.4%. Septic shock was diagnosed in 4% of cases and it could be considered both a predisposing condition and a consequence of CDI. Regarding the 8.5% of CD infected patients with sepsis (bacteremia or candidemia), no data are available on the timing of sepsis.

Regarding comorbidities that are not risk factors for CDI, the most frequently reported were diabetes in 11.5% of cases and hypertension in 10%.

Districts with the greatest number of hospitals reporting CDI were Catania, Palermo, and Messina (with 20, 14, and 13 hospitals, respectively), which are the biggest districts in the island with the greatest number of inhabitants and Infectious Diseases units. 86% of CDI cases derived from level III and II hospitals, which accounted for around 49% of reporting hospitals. LTCFs and level I hospitals accounted for 50% of reporting hospitals but reported just 13% of CDI cases.

The higher number of CDI cases in level III and II hospitals probably derives from a greater severity of hospitalized patients, who experience longer hospitalizations and a greater number of infectious complications (including CDI). Furthermore, it probably also derives from more under-reporting from LTCFs, and level I hospitals, where there is scarce opportunity for ID consults and where physicians may have inadequate skills or backgrounds regarding CDI diagnosis.

Regarding the distribution of CDI cases in different wards, in our population, 86.8% were reported in Internal Medicine/Geriatrics, Infectious Diseases (ID) Units, Long-term care facilities (LTCFs)/Rehabilitation centre and General Surgery Units (51, 22.5, 7.9, and 5.4%, respectively). The higher incidence found in the above wards may be due to the older age and comorbidities of patients in Internal Medicine/Geriatrics, to longer hospitalizations, particularly in LTCFs, and to recent abdominal surgery in General Surgery, as has already been described in the literature [14, 25].

Indeed, in the USA, the highest incidence of CDI is described in LTCFs, even if the real burden is unknown and likely much higher since LTCFs often do not report CDI data [5, 26, 27]. Higher incidence in ID units may be partially explained by a greater rate of diagnosis due to much higher awareness of the problem by ID specialists.

In our population, considering all the wards, we found a mean length of stay of 20 days. CDC reports a median length of stay of 8 days [28]. No data on this are available in Europe. We analyzed the mean length of stay in different groups; for male vs female patients and for patients who died with CDI during hospitalization and those who did not, and we found no significant differences (*p* value 0.1 and 0.22, respectively).

Even if we are neither able to establish the precise timing of CDI during hospitalization nor the relationship between CDI and prolonged in-hospital stay, we are aware that such a large length of stay represents a risk factor for developing CDI. Regarding the distribution of CDI in different districts and hospitals, an increasing trend over the decade was documented in the districts of Catania, Palermo, Messina, Siracusa and Ragusa, which reported 98.5% of total CDI cases during the decade. On the other hand, in the districts of Caltanissetta, Agrigento, Enna and Trapani the expected increasing trend was not noted, and very few CDI cases were reported during the whole decade (1.5% of total CDI cases).

This reveals that CDI is an emerging problem but also that an increased awareness by clinicians is probably needed in these last districts.

Catania, Palermo and Messina are the biggest districts with the highest number of inhabitants, reporting hospitals and ID units. Siracusa is the 5th most populous Sicilian district and reported 152 CDI cases over the decade, while the district of Agrigento, which is the 4th most populous, reported just 3 CDI cases in the same period. It is likely the majority of underreporting derives mainly from the districts of Ragusa, Caltanissetta, Agrigento, Enna and Trapani. Indeed, Ragusa reported 51 cases from 3 different hospitals, Caltanissetta and Agrigento just 6 and 3 cases from 2 and 4 hospitals, respectively, and Enna and Trapani just 1 case from 1 hospital each. This supports the need to invest resources to raise awareness of CDI by clinicians above all in the cited districts. The case-fatality rate in patients with CDI revealed an increasing trend over the 10 years, with the minimum recorded in 2009 (5.2%) and the maximum in 2017 (14.2%), and a statistically significant difference in mean age at death recorded between female and male patients, higher in females compared to males (80.9 vs 74.5). In Europe, the most recent updated data report in-hospital mortality by any cause of 20.7% and an attributable mortality rate around 3% [4].

The attributable mortality rate described in the US was < 2% and 4.5–5.7% before and after 2000, respectively [5]. In particular, it was 5.7% in 2017 considering just hospitalized patients with CDI [3].

### Study limits

Regarding “Materials and methods”: first of all, it is a retrospective study. In spite of utilizing a regional-wide database, it lacks clinical characteristics of patients, including laboratory and radiologic data, and therapy (antibiotics, proton pump inhibitors and H2 blockers, in particular). It also lacks data on the main reason for hospital admissions and timing of coinfections. Microbiological means for CDI diagnosis are not specified, so we are unable to establish how many CDI cases were diagnosed following the ECDC recommendations. We have no data on *C. difficile* therapy, nor on follow-up and recurrences. Indeed, the regional database provides anonymous data, so it is neither possible to establish if some patients experienced a CDI recurrence during the same admission nor if the same patients were readmitted for a recurrence.

As our database captures neither cause of death nor specific timing of infection, we are not able to estimate CDI-related mortality, or the role of CDI on potentially extending the length of stay. Also, we used ICD-9 00,845 codes which may not be highly sensitive in capturing *C. difficile* infection in inpatient datasets. Indeed, in the SDO a maximum of six diagnoses can be inserted, so for patients with multiple comorbidities and a long length of stay, it could be possible that CDI diagnosis had not been entered. In addition, this code refers to CDI with or without complication and there is no specific code for CDI-related complication.

We believe that the limits of our study are balanced by the large sample size with data spanning over 10 years and that our study adds paramount epidemiological information on CDI.

## Conclusions

In the last decade in Sicily, 1139 CDI cases were reported to the Regional Healthcare Department, with a dramatic increase in incidence between 2009 and 2019. The majority of cases occurred in the metropolitan areas and in the biggest hospitals, with a slightly higher incidence in women, who, in our population were older both considering the total number of cases and just the patients who died. The mean length of stay was 20 days with no difference between female and male patients, nor between patients who died and who did not. Concomitant infections were documented in at least 20.9% of cases. The mean case fatality rate was 8.3% over the 10 years. Increase in incidence is striking, despite the likely under-reporting, so an active and high-quality surveillance system and shared protocols are needed.

CDI underdiagnosis because of absence of clinical suspicion and under-reporting require initiatives to raise physicians’ awareness of CDI.

## Supplementary Information

Below is the link to the electronic supplementary material.

Supplementary file1 (DOCX 102 KB)

## Data Availability

Available if required.

## Abbreviations

CDI: *C. difficile* Infection
GDH: Glutamate dehydrogenase
EIA: Enzyme immunoassay
HAI: Hospital acquired infection
HCAI: Health care-associated infection
LTCF: Long-term care facilities
IBD: Inflammatory bowel disease
SOT: Solid organ transplant
HSCT: Haematological stem cell transplantation
ECDC: European Centre for Disease Prevention and Control
ISS: Italian Superior Health Institute
CCM: Italian Centre for Diseases Control and Prevention
DASOE: Dipartimento per le Attività Sanitarie e Osservatorio Epidemiologico
SDO: National standardized discharge form
ICD-9-CM: International Classification of Diseases, 9th revision, Clinical Modification
MDRO: Multidrug-resistant microorganisms
CRKP: Carbapenem-resistant *K. pneumoniae*
ECDIS-Net project: ECDC-funded “Supporting capacity building for surveillance of *C. difficile* infections
ESAC-Net: European Surveillance of Antimicrobial consumption network
DDD: Defined daily doses

## References

[1] Data Portal | HAI | CDC. https://www.cdc.gov/hai/data/portal/index.html. Accessed 1 Feb 2020.

[2] Magill SS, O’Leary E, Janelle SJ (2018). Changes in prevalence of health care-associated infections in US Hospitals. N Engl J Med.

[3] WHERE INFECTIONS HAPPEN. http://www.cdc.gov/DrugResistance/Biggest-Threats.html. Accessed 20 Mar 2021.

[4] ECDC. Annual epidemiological report for 2016 *Clostridium difficile* infections. 2018.

[5] McDonald LC, Gerding DN, Johnson S (2018). Clinical practice guidelines for *Clostridium difficile* infection in adults and children: 2017 update by the Infectious Diseases Society of America (IDSA) and Society for Healthcare Epidemiology of America (SHEA). Clin Infect Dis.

[6] Quan KA, Yim J, Merrill D (2018). Reductions in *Clostridium difficile* infection (CDI) rates using real-time automated clinical criteria verification to enforce appropriate testing. Infect Control Hosp Epidemiol.

[7] McDonald LC, Coignard B, Dubberke E, Song X, Horan T, Kutty PK (2007). Recommendations for surveillance of *Clostridium difficile*-associated disease. Infect Control Hosp Epidemiol.

[8] Pépin J, Valiquette L, Alary ME (2004). *Clostridium difficile*-associated diarrhea in a region of Quebec from 1991 to 2003: a changing pattern of disease severity. CMAJ.

[9] McDonald LC, Killgore GE, Thompson A (2005). An epidemic, toxin gene-variant strain of *Clostridium difficile*. N Engl J Med.

[10] Kuijper EJ, Barbut F, Brazier JS (2008). Update of *Clostridium difficile* infection due to PCR ribotype 027 in Europe, 2008. Euro Surveill.

[11] Featuring, Survey of diagnostic and typing capacity for *Clostridium difficile* infection in Europe, 2011 and 2014. Standardised surveillance of *Clostridium difficile* infection in European acute care hospitals: a pilot study, 2013. Enhanced surveillance of *Clostridium difficile* infection occurring outside hospital *Clostridium difficile* infection in Europe. Published online 2016. 10.2807/1560.

[12] Ka D, Cm L, Gl D (2014). Underdiagnosis of *Clostridium difficile* across Europe: the European, multicentre, prospective, biannual, point-prevalence study of *Clostridium difficile* infection in hospitalised patients with diarrhoea (EUCLID). Lancet Infect Dis.

[13] Masucci L, Nicolotti N, Graffeo R (2019). *Clostridium difficile*: trend in an Italian Tertiary Care Hospital during fifteen years, 2002–2016. Minerva Med.

[14] Mancini A, La Vigna G, Puciarelli S, Lombardi FE, Barocci S (2018). A three-year study entailing molecular characterization and epidemiology of *Clostridium difficile* in an Italian tertiary care hospital. Le Infez Med..

[15] Granata G, Petrosillo N, Adamoli L (2021). Prospective study on incidence, risk factors and outcome of recurrent *Clostridioides difficile* infections. J Clin Med.

[16] Cataldo MA, Granata G, D’Arezzo S (2021). Hospitalized patients with diarrhea: rate of *Clostridioides difficile* infection underdiagnosis and drivers of clinical suspicion. Anaerobe.

[17] Sistema informativo malattie infettive, Simi. https://www.epicentro.iss.it/infettive/sorveglianza. Accessed 31 Jan 2020.

[18] Infezioni correlate all’assistenza news. https://www.epicentro.iss.it/infezioni-correlate/aggiornamenti. Accessed 31 Jan 2020.

[19] Antimicrobial consumption—annual epidemiological report for 2019. https://www.ecdc.europa.eu/en/publications-data/surveillance-antimicrobial-consumption-europe-2019. Accessed 20 Mar 2021.

[20] Hensgens MPM, Goorhuis A, Dekkers OM, Kuijper EJ (2012). Time interval of increased risk for *Clostridium difficile* infection after exposure to antibiotics. J Antimicrob Chemother.

[21] Pépin J, Saheb N, Coulombe M-A (2005). Emergence of fluoroquinolones as the predominant risk factor for *Clostridium difficile*-associated diarrhea: a cohort study during an epidemic in Quebec. Clin Infect Dis.

[22] Carbapenem-resistant Enterobacteriaceae (CRE). https://www.ecdc.europa.eu/en/publications-data/directory-guidance-prevention-and-control/prevention-and-control-infections-1. Accessed 26 Feb 2021.

[23] La scheda di dimissione ospedaliera (SDO). http://www.salute.gov.it/portale/temi/p2_6.jsp?lingua=italiano&id=1232&area=ricoveriOspedalieri&menu=vuoto. Accessed 30 Jan 2020.

[24] ICD—ICD-9-CM—International Classification of Diseases, Ninth Revision, Clinical Modification. https://www.cdc.gov/nchs/icd/icd9cm.htm. Accessed 31 Jan 2020.

[25] Jump RLP (2013). *Clostridium difficile* infection in older adults. Aging Health.

[26] Hunter JC, Mu Y, Dumyati GK (2016). Burden of nursing home-onset *Clostridium difficile* infection in the United States: estimates of incidence and patient outcomes. Open forum Infect Dis.

[27] Simor AE, Bradley SF, Strausbaugh LJ, Crossley K, Nicolle LE (2002). *Clostridium difficile* in long-term-care facilities for the elderly. Infect Control Hosp Epidemiol.

[28] 2018 annual report for the emerging infections program for *Clostridioides difficile* infection | Emerging Infections Program | HAI | CDC. https://www.cdc.gov/hai/eip/Annual-CDI-Report-2018.html. Accessed 9 Mar 2021.

