# Correction to: An emergent infectious disease: *Clostridioides difficile* infection hospitalizations, 10-year trend in Sicily

**DOI:** 10.1007/s15010-021-01700-y

**Published:** 2021-10-11

**Authors:** Alice Annalisa Medaglia, Sergio Buffa, Claudia Gioè, Silvia Bonura, Raffaella Rubino, Chiara Iaria, Claudia Colomba, Antonio Cascio

**Affiliations:** 1 Infectious and Tropical Disease Unit, AOU Policlinico “P. Giaccone”, Palermo, Italy; 2 Dipartimento per le Attività Sanitarie e Osservatorio Epidemiologico (DASOE), Palermo, Italy; 3 Infectious Diseases Unit, ARNAS Civico, Palermo, Italy; 4 Department of Health Promotion, Mother and Child Care, Internal Medicine and Medical Specialties, Infectious Diseases Unit, University of Palermo, Palermo, Italy

## Correction to: Infection 10.1007/s15010-021-01683-w

In the “Results” section of this article, the sentence “Among male patients, 0.11% died (54/478 pts), and among female patients, 0.09% died (62/663 pts).” should instead have read “Among male patients, 11% died (54/478 pts), and among female patients, 9% died (62/663 pts).”.

The original article has been corrected.

