# Supplementary material for: An emergent infectious disease: Clostridioides difficile infection hospitalizations, 10-year trend in Sicily
Source: Infection. 2021 Sep 8;49(6):1221–9. doi: 10.1007/s15010-021-01683-w (PMC8613107; doi:10.1007/s15010-021-01683-w)
Supplement: Supplementary file 1 — Supplementary file1 (DOCX 102 KB) [file 15010_2021_1683_MOESM1_ESM.docx]

Supplemental materials

Most of the cases where provided from 7 hospitals: hospital 1 of Siracusa (110 cases), hospital 1 of Messina (95 cases), hospital 1, 2, 3, and 4 of Catania (respectively 88, 82, 78, 62), and hospital 1 of Palermo (60 cases). See the graphic for the details on number of cases for each of the 21 hospitals which reported at least 10 cases. All the other 44 hospitals reported less than 10 cases each, so the detail for these is not graphed.


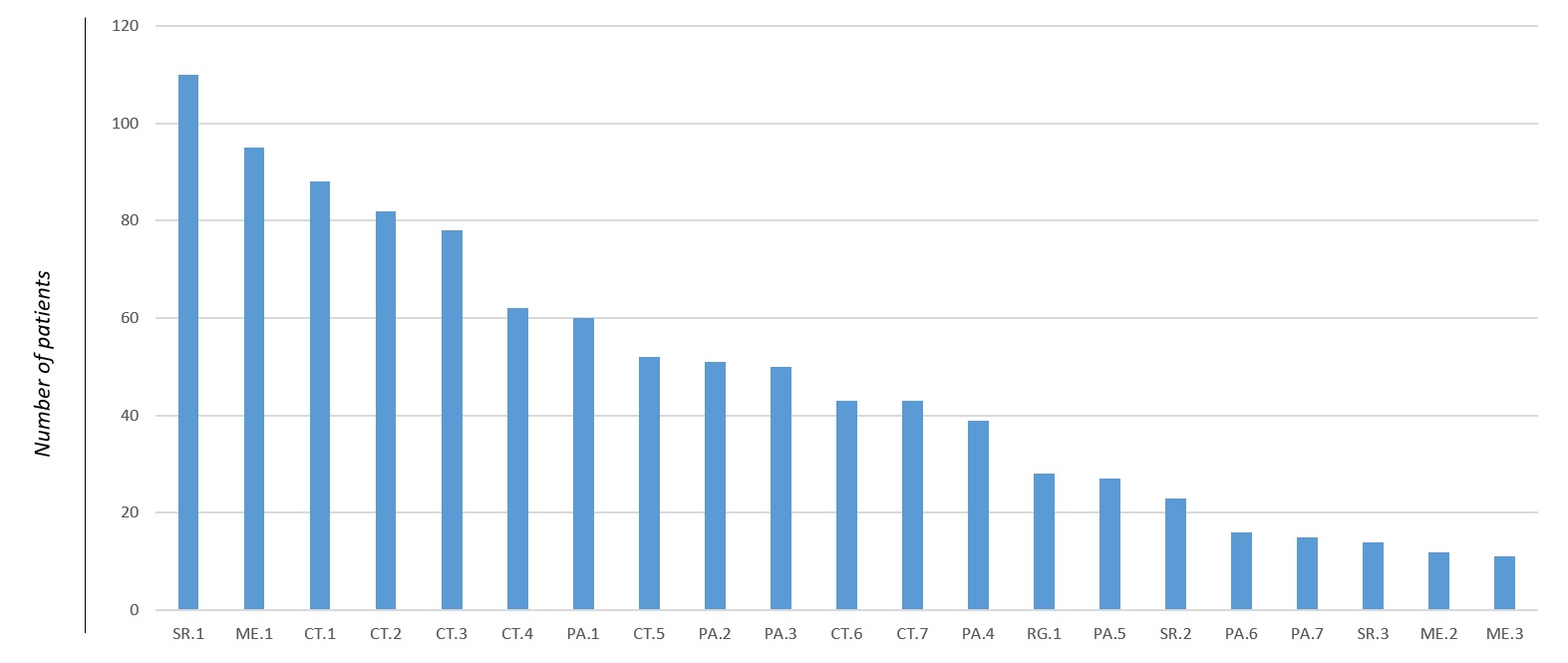


Graphic 1 of Supplemental materials – Number of CDI cases per hospital (detail graphed just for the 21 hospitals reporting at least 10 cases each)
